# Increased mean diffusivity of the caudal motor SNc identifies patients with REM sleep behaviour disorder and Parkinson’s disease

**DOI:** 10.1038/s41531-024-00731-0

**Published:** 2024-06-29

**Authors:** Erind Alushaj, Dimuthu Hemachandra, Hooman Ganjavi, Ken N. Seergobin, Manas Sharma, Alia Kashgari, Jennifer Barr, William Reisman, Ali R. Khan, Penny A. MacDonald

**Affiliations:** 1 Department of Neuroscience, Schulich School of Medicine and Dentistry, Western University, London, ON, Canada; 2 Western Institute for Neuroscience, Western University, London, ON, Canada; 3 Robarts Research Institute, Western University, London, ON, Canada; 4 School of Biomedical Engineering, Western University, London, ON, Canada; 5 Department of Psychiatry, Western University, London, ON, Canada; 6 Department of Radiology, Western University, London, ON, Canada; 7 Department of Clinical Neurological Sciences, Western University, London, ON, Canada; 8 Department of Medicine, Respirology Division, Western University, London, ON, Canada; 9 Department of Medical Biophysics, Western University, London, ON, Canada

**Keywords:** Diagnostic markers, Neurodegeneration

## Abstract

Idiopathic rapid eye movement sleep behaviour disorder (iRBD)—a Parkinson’s disease (PD) prodrome—might exhibit neural changes similar to those in PD. Substantia nigra pars compacta (SNc) degeneration underlies motor symptoms of PD. In iRBD and early PD (ePD), we measured diffusion MRI (dMRI) in the caudal motor SNc, which overlaps the nigrosome-1—the earliest-degenerating dopaminergic neurons in PD—and in the striatum. Nineteen iRBD, 26 ePD (1.7 ± 0.03 years), and 46 age-matched healthy controls (HCs) were scanned at Western University, and 47 iRBD, 115 ePD (0.9 ± 0.01 years), and 56 HCs were scanned through the Parkinson’s Progression Markers Initiative, using 3T MRI. We segmented the SNc and striatum into subregions using automated probabilistic tractography to the cortex. We measured mean diffusivity (MD) and fractional anisotropy (FA) along white-matter bundles and subregional surfaces. We performed group-level and classification analyses. Increased caudal motor SNc surface MD was the only iRBD-HCs and ePD-HCs difference replicating across datasets (*p*_adj_ < 0.05). No iRBD-ePD differences emerged. Caudal motor SNc surface MD classified patient groups from HCs at the *single-subject level* with good-to-excellent balanced accuracy in an independent sample (0.91 iRBD and 0.86 iRBD and ePD combined), compared to fair performance for *total* SNc surface MD (0.72 iRBD and ePD). Caudal motor SNc surface MD correlated significantly with MDS-UPDRS-III scores in ePD patients. Using dMRI and automated segmentation, we detected changes suggesting altered microstructural integrity in iRBD and ePD in the nigrostriatal *sub*region known to degenerate first in PD. Surface MD of the caudal motor SNc presents a potential measure for inclusion in neuroimaging biomarkers of iRBD and PD.

## Introduction

Parkinson’s disease (PD) is a progressive, neurodegenerative illness causing both motor and non-motor symptoms^1^. PD lacks disease-modifying therapies or a cure^2^. Accurate, sensitive, and accessible measures of PD presence and progression, especially in its earliest stages, would facilitate drug discovery and clinical management^3,4^.

Dopamine transporter (DaT) imaging is helpful, especially a negative result that considerably reduces the probability of idiopathic PD^5^. However, DaT is expensive, which is a disadvantage for both research and clinical applications. Furthermore, it is rather inaccessible to clinicians outside of specialised movement disorder clinics^5–8^. These clinicians will increasingly participate in the diagnosis and management of PD^3^. MRI is lower cost and more available to clinical generalists. MRI has shown promise in distinguishing PD and age-matched healthy control (HC) groups^9,10–12^ and in tracking PD progression^13,14^. In this study, we focused on the potential of MRI to investigate the earliest stages of PD to provide diagnostic and progression biomarkers of disease.

The cardinal motor symptoms of PD—bradykinesia, rigidity, and resting tremor—are attributed to the degeneration of dopamine-producing neurons in the substantia nigra (SN) pars compacta (SNc), and the consequent dopamine depletion of the caudal motor subregion of the striatum^9,15–17^. Iron^10,12,18^, neuromelanin^19,20^, and diffusion^21^ MRI measures reveal abnormalities in the SN/SNc and striatum in PD. Few studies, however, present MRI measures that accurately identify PD patients at the individual level. This is needed for these measures to perform much-needed diagnostic tests of PD to facilitate therapeutic innovation and clinical management. MRI measures track PD evolution^22,23^, suggesting utility in the discovery of disease-modifying therapies.

By the time that PD is clinically diagnosed, the preponderance of SNc dopaminergic neurons have already degenerated with greater sparing in the ventral tegmental area^10,24^. Idiopathic rapid eye movement (REM) sleep behaviour disorder (iRBD) is a sleep disorder in which patients act out their dreams due to failure of normal muscular paralysis during dreaming^25^. Within 12 years of iRBD onset, nearly 75% of iRBD patients will develop a neurodegenerative alpha-synucleinopathy^26^. iRBD is considered prodromal PD because more than 50% of these patients will eventually develop motor symptoms of PD, or phenoconvert to PD^27–29^. Furthermore, normal cognition in iRBD strongly biases phenoconversion to PD, rather than to another alpha-synucleinopathy^26^, providing a unique opportunity to study neural changes preceding motor symptoms and to uncover quantifiable indicators—biomarkers—of prodromal PD.

In the sparse literature, some studies^30,31^ but not others reveal MRI changes in SN/SNc and/or striatum in iRBD patients, which mimic neural alterations in PD^18,32,33^. This is coherent with the notion that iRBD and PD are stages along a disease continuum. These changes include increased iron in SN/SNc^34,35^, increased fractional anisotropy^30^ in the SN, and decreased mean diffusivity^31^ in the SNc in iRBD relative to HCs. dMRI measures water diffusion that normally moves preferentially along axonal trajectories in vivo^36^. FA is an index of directional diffusion whereas MD measures overall diffusion^37^, with decreases in FA and increases in MD signalling abnormalities in brain microstructural integrity. This small and conflicting literature suggests the potential of MRI as a tool for investigating iRBD. This warrants greater investigation, however, particularly toward estimating the sensitivity of MRI to appropriately detect individuals with PD at the single-subject level, let alone prodromal PD patients. Diagnostic biomarkers of PD or prodromal PD need to accurately detect individual patients.

In the current study, we investigated neural changes in iRBD relative to ePD and HCs using dMRI measures in a) *subregions* of the VTA/SNc and striatum, versus b) *total* VTA/SNc and striatum. We measured dMRI features from both white matter and grey matter—bundle FA/MD and surface FA/MD, respectively. Our VTA/SNc and striatal segmentations were premised on the differential susceptibility of *subregions* to PD^9,18^, and evidence of functional heterogeneity of the striatum^38^. We used probabilistic tractography to parcellate the VTA/SNc into four subregions and the striatum into six subregions—determined by preferential connectivity to Harvard-Oxford atlas-defined cortical subregions. We expected that the sensitivity of dMRI to detect changes in prodromal PD would be increased by isolating measures of (a) earliest-degenerated SNc subregion (i.e. ventrolateral subregion/nigrosome-1), termed the caudal motor (CM) SNc and (b) most dopamine-depleted striatal subregion, termed the CM striatum. Using this automated approach to parcellate the striatum, we previously discovered PD-HC volume/shape differences in CM but not total striatum^9^ and good sensitivity with CM striatal iron to detect individuals with PD^18^. The focus, here, was to investigate the potential of dMRI in segmented versus total VTA/SNc and striatum to a) study neural changes in cognitively intact iRBD/premotor PD relative to ePD and HCs, and b) provide diagnostic, phenoconversion, and progression biomarkers.

Validated diagnostic biomarkers of prodromal *PD—*objective measures identifying individuals not yet manifesting motor signs but who will develop PD—could facilitate recruitment of patients into clinical trials at stages when disease-modifying potential of therapies is anticipated to be maximal due to greater neural substrate to protect^26,39^. Not all iRBD/RBD patients represent prodromal-PD/alpha-synucleinopathy^39,40^ and hence an objective test to distinguish these patients will be important. The availability of quantifiable boundaries between iRBD and PD—phenoconversion biomarkers—are highly-prised targets for neuroprotective therapy development, but seems unlikely given preponderant evidence suggesting these are manifestations of disease along a spectrum^39^. Finally, similar to phenoconversion biomarkers, PD progression biomarkers could provide objective endpoints for clinical trials. PD is highly heterogenous, and clinical symptoms and signs vary remarkably within the same patient on repeat testing, posing significant challenges for establishing the efficacy of disease-modifying therapies^39^. Objective measures sensitive to PD onset or progression could expedite screening of therapies for their potential to prevent/delay PD or its advance, requiring smaller groups of patients, followed for shorter durations, to establish efficacy. Only those treatments screened positively could then be investigated in costly trials that include clinical endpoints^41^.

To identify the most promising dMRI biomarkers of prodromal *PD diagnosis* and of *iRBD-to-PD phenoconversion*, we selected significant iRBD-HCs and iRBD-PD differences, respectively, that (a) survived conservative Bonferroni correction for multiple comparisons and (b) replicated in samples tested i) at Western University (i.e. the local Western dataset) and ii) through the multicentred Progressive Parkinson’s Markers Initiative (PPMI). These measures were next investigated for their potential to distinguish iRBD patients from a) HCs and b) PD, using receiver operating characteristic (ROC) curve-area under the curve (AUC) analyses, with cross-validation, and tested in independent hold-out sets. Finally, the most promising dMRI measures distinguishing iRBD-HCs and ePD-HCs will be correlated with the MDS-UPDRS III (motor subscale) to assess their potential as *progression biomarkers*.

## Results

### Demographics

#### Local Western

For the local Western dataset, groups did not differ significantly in Age, *F(*2,87) = 2.93, *p* = 0.06, and years of Education, *F*(2,84) = 2.07, *p* = 0.13. Predictably, there were significant differences in Sex, *χ*^2^(2) = 7.39, *p* = 0.03, due to the male-dominant patient groups. There were significant differences in total Montreal Cognitive Assessment (MoCA) scores, *F*(2,87) = 3.67, *p* = 0.03 but no significant differences in the pairwise comparisons (HC versus iRBD *p* = 0.07, HC versus ePD *p* = 0.19, iRBD versus ePD *p* = 0.90). Only participants with MoCA > 23 were included in the study. The RBD questionnaire Hong Kong (RBDQ-HK) scores, *H*(2) = 16.9, *p* < 0.001, were predictably higher in iRBD than HCs and ePDs (Table 1). Finally, higher MDS-UPDRS III scores for ePD patients produced a significant omnibus effect, *H*(2) = 38.3, *p* < 0.001, with ePD-HCs *p*_adj_ < 0.001, ePD-iRBD *p*_adj_ < 0.001 differences, but no significant iRBD-HCs difference *p*_adj_ = 0.19 (Table 1).

**Table 1:** Demographic and clinical information for local Western participants

	iRBD	ePD	HC	*p* value
Sex (F:M)	4:15	9:17	25:20	**<0.05**
Age (years)	67.1 ± 1.5	67.4 ± 1.5	63.6 ± 1.1	0.06
Education (years)	15.1 ± 0.6	14.6 ± 0.6	16.0 ± 0.4	0.13
MoCA Total (/30)	26.6 ± 0.6	26.9 ± 0.5	28.0 ± 0.3	**<0.05**
Disease Duration (years)	3.4 ± 0.6	1.7 ± 0.3	-	-
MDS-UPDRS-III (/132)	2.9 ± 0.5	33.2 ± 2.9	0.8 ± 0.4	**<0.001**
RBDQ-HK (/100)	43.0 ± 4.7	25.8 ± 5	14.1 ± 2.3	**<0.001**

Cells show the ratio of female to male sex, age in years, education in years, MoCA total scores, disease duration in years, MDS-UPDRS-III and RBDQ-HK. Averages are reported as means ± standard error mean.

#### PPMI

For the PPMI dataset, there were no significant differences in age, *H*(2) = 2.20, *p* = 0.33, years of education, *F*(2,192) = 1.17, *p* = 0.31, and MoCA Total scores, *H*(2) = 1.22, *p* = 0.54. iRBD patients scored higher on RBDSQ, *H*(2) = 92, *p* < 0.001^42^. iRBD mean disease duration was 2.7 ± 1.3 years (data available for only 10/ 47, as this variable is now deprecated). Male-dominant patient groups produced differences in Sex, *χ*^2^(2) = 7.69, *p* = 0.02. MDS-UPDRS-III scores differed for Group, *H*(2) = 149, *p* < 0.001, with ePD>HCs (*p*_adj_ < 0.001) and iRBD>HCs (*p*_adj_ < 0.001). iRBDs scored 3.33/132 points higher on average than HCs (*p*_adj_ = 0.04), indicating no clinically meaningful difference (Table 2).

**Table 2:** Demographic and clinical information for PPMI participants

	iRBD	ePD	HC	*p* value
Sex (F:M)	11:37	44:71	21:20	**<0.05**
Age (years)	67.7 ± 0.6	66.4 ± 0.6	66.6 ± 1.1	0.33
Education (years)	16.2 ± 0.6	15.3 ± 0.3	15.2 ± 0.5	0.31
MoCA Total (/30)	27.5 ± 0.3	27.5 ± 0.2	28.0 ± 0.2	0.54
Disease Duration (years)	2.7 ± 1.3^a^	0.9 ± 0.1	-	-
MDS-UPDRS-III (/132)	4.0 ± 0.6	22.8 ± 1.0	0.66 ± 0.2	**<0.001**
Hoehn & Yahr	0.1 ± 0.06	1.7 ± 0.1	0.0 ± 0.02	-
RBDSQ (/13)	10.3 ± 0.4	4.5 ± 0.3	2.2 ± 0.3	**<0.001**

^a^Disease duration data were only available for 10 of the iRBD patients due to PPMI variable deprecation.

Cells show the ratio of female to male sex, age in years, education in years, MoCA total scores, disease duration in years, MDS-UPDRS-III and RBDSQ. Averages are reported as means ± standard error mean.

### Results of ANCOVAs on dMRI features

The adjusted *p* values of the ANCOVAs conducted on the eight features across the two unparcellated regions and ten parcellated subregions of the VTA, SNc and striatum are summarised in Table 3. Group differences were only revealed for both the Western dataset and the multicentred PPMI dataset in the CM SNc surface MD, which overlaps the nigrosome-1. Pairwise comparisons revealed significant differences between iRBD>HCs and ePD>HCs. iRBD and ePD were not significantly different, as detailed below.

**Table 3:** Summary of VTA, SNc, and striatum unparcellated and parcellated subregion ANCOVAs in participants from local Western and PPMI data

	Local Western				PPMI			
Region	Main Effect of Group from ANCOVA (RBD vs ePD vs HC)							
**VTA, SNc**	Bundle MD	Surface MD	Bundle FA	Surface FA	Bundle MD	Surface MD	Bundle FA	Surface FA
Unparcellated SNc	-	-	0.01	-	-	-	-	-
Limbic	-	-	-	-	-	-	-	-
Executive	-	-	0.01	0.004	-	-	-	-
Rostral Motor	-	-	<0.001	-	-	-	-	-
Caudal Motor	-	**<0.001**	<0.001	-	-	**0.003**	-	-
**Striatum**	Bundle MD	Surface MD	Bundle FA	Surface FA	Bundle MD	Surface MD	Bundle FA	Surface FA
Unparcellated	-	0.03	-	-	-	-	-	-
Limbic	-	-	-	-	-	-	-	-
Executive	-	<0.001	-	-	-	-	-	-
Rostral Motor	-	0.01	-	-	-	-	-	-
Caudal Motor	-	-	-	-	-	-	-	-
Parietal	-	<0.001	-	-	-	-	-	-
Occipital	-	<0.001	-	-	-	-	-	-
	**post-hoc** **comparisons (RBD vs HC)**							
**VTA, SNc**	Bundle MD	Surface MD	Bundle FA	Surface FA	Bundle MD	Surface MD	Bundle FA	Surface FA
Unparcellated SNc	-	-	0.03	-	-	-	-	-
Limbic	-	-	-	-	-	-	-	-
Executive	-	-	<0.001	<0.001	-	-	-	-
Rostral Motor	-	-	<0.001	-	-	-	-	-
Caudal Motor	-	**<0.001**	<0.001	-	-	**0.002**	-	-
**Striatum**	Bundle MD	Surface MD	Bundle FA	Surface FA	Bundle MD	Surface MD	Bundle FA	Surface FA
Unparcellated	-	0.03	-	-	-	-	-	-
Limbic	-	-	-	-	-	-	-	-
Executive	-	<0.001	-	-	-	-	-	-
Rostral Motor	-	0.01	-	-	-	-	-	-
Caudal Motor	-	-	-	-	-	-	-	-
Parietal	-	<0.001	-	-	-	-	-	-
Occipital	-	<0.001	-	-	-	-	-	-
	**post-hoc** **comparisons (ePD vs HC)**							
**VTA, SNc**	Bundle MD	Surface MD	Bundle FA	Surface FA	Bundle MD	Surface MD	Bundle FA	Surface FA
Unparcellated SNc	-	-	-	-	-	-	-	-
Limbic	-	-	-	-	-	-	-	-
Executive	-	-	-	<0.001	-	-	-	-
Rostral Motor	-	-	-	-	-	-	-	-
Caudal Motor	-	**0.004**	-	-	-	**<0.001**	-	-
**Striatum**	Bundle MD	Surface MD	Bundle FA	Surface FA	Bundle MD	Surface MD	Bundle FA	Surface FA
Unparcellated	-	-	-	-	-	-	-	-
Limbic	-	-	-	-	-	-	-	-
Executive	-	-	-	-	-	-	-	-
Rostral Motor	-	-	-	-	-	-	-	-
Caudal Motor	-	-	-	-	-	-	-	-
Parietal	-	-	-	-	-	-	-	-
Occipital	-	-	-	-	-	-	-	-

Cells show adjusted *p* values of the main effect of Group and post-hoc comparisons for the dMRI features in the VTA, SNc and striatum regions of interest of the local Western and PPMI datasets following Bonferroni correction. Surface MD in the caudal motor SNc was significant in both datasets.

### VTA and SNc: Mean bundle MD and FA

#### Local Western dataset

The mean FA of the unparcellated SNc revealed a significant main effect of Group, *H*(2) = 12.8, *p*_adj_ = 0.04, with iRBD patients showing lower values than HCs, *z* = 3.4, *p*_adj_ = 0.03. ANCOVAs were significant for the main effect of Group for mean FA in the SNc executive, *H*(2) = 17.4, *p*_adj_ = 0.01, rostral motor, *H*(2) = 17.5, *p*_adj_ = 0.01, and CM subregions, *H*(2) = 29.6, *p*_adj_ = 0.001. iRBD patients evidenced lower mean bundle FA compared to HCs in the executive, *z* = 4.2, *p*_adj_ < 0.001, rostral motor, *z* = 4.1, *p*_adj_ < 0.001, and CM subregions, *z* = 4.9, *p*_adj_ < 0.001. iRBD patients also showed lower mean bundle FA compared to ePDs in the executive, *z* = 2.8, *p*_adj_ = 0.01, rostral motor, *z* = 3.2, *p*_adj =_ 0.004, and CM subregions, *z* = 4.9, *p*_adj_ < 0.001. All other Bonferroni-corrected contrasts failed to reach significance.

#### PPMI dataset

No main effects of mean bundle MD and FA reached significance.

### VTA and SNc: mean surface MD and FA

#### Local Western dataset

**Fig. 1:**
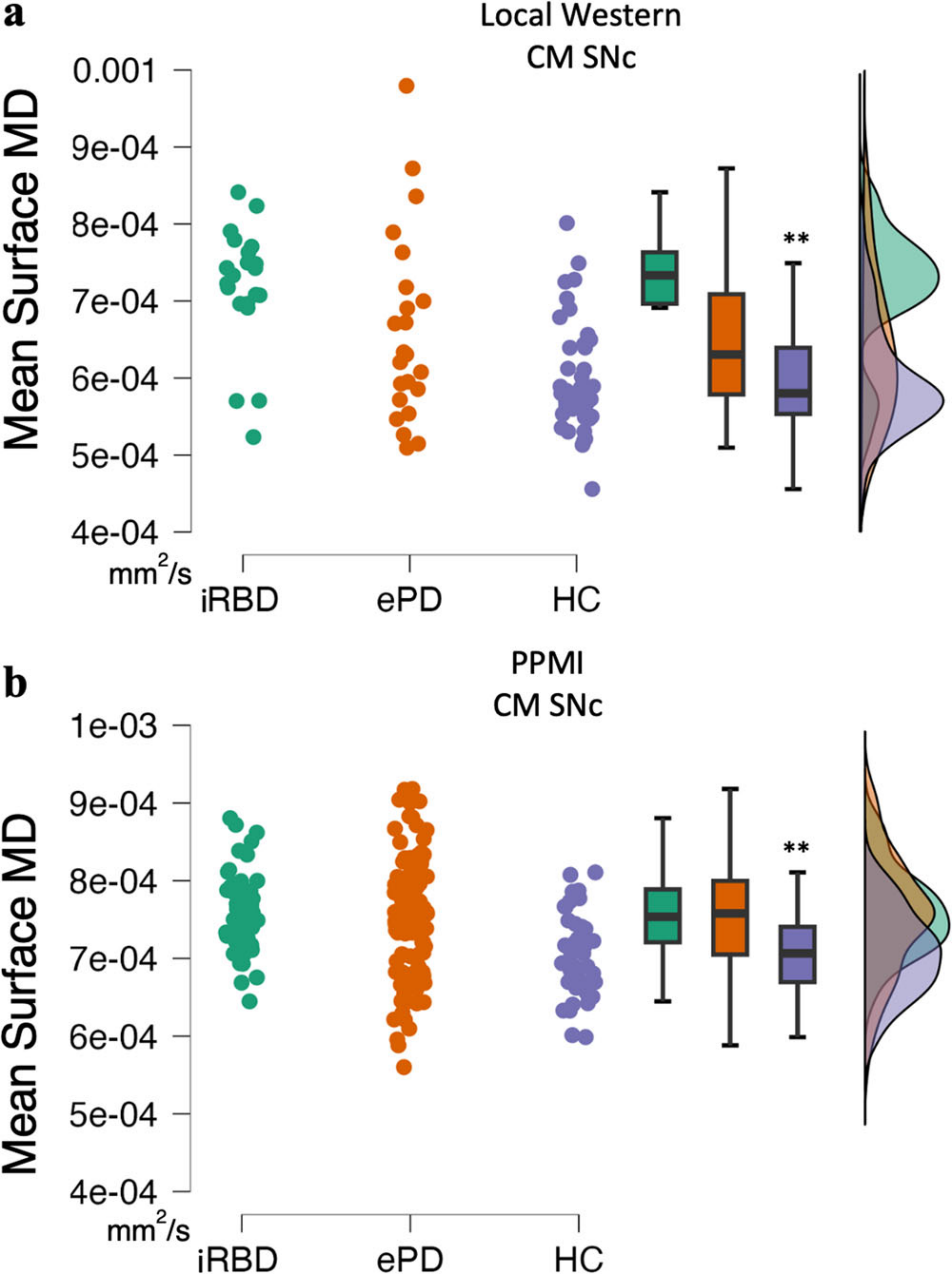
CM SNc mean surface MD for each Group: Western and PPMI datasets presented separately. CM SNc mean surface MD of the **a** local Western and **b** PPMI data for iRBD patients, ePD patients and HCs. Data were shown as scatterplots and boxplots for the CM SNc mean surface MD in mm^2^/s. **a** Local Western iRBD and ePD patients had higher mean surface MD relative to HCs. **b** PPMI iRBD and ePD patients showed higher mean surface MD compared to HCs. Local Western: n_RBD_ = 19, n_ePD_ = 26, n_HC_ = 45. PPMI: n_RBD_ = 48, n_ePD_ = 115, n_HC_ = 41. **p_adj_ < 0.01.

There was a significant main effect of Group in the measure of surface MD in the CM SNc, *F*(2,85) = 9.6, *MSe* = 9E-9, *p*_adj_ < 0.001, *η*²_p_ = 0.18, with HCs having lower mean surface MD compared to iRBD patients, *t*(62) = 3.9, *p*_adj_ < 0.001, and ePD patients, *t*(69) = 3.3, *p*_adj_ = 0.004. There was no difference between iRBD and ePD in this measure (Fig. 1a).

The mean surface FA in the SNc executive subregion showed a significant main effect of Group, *F*(2,85) = 12.0, *MSe* = 3E-3, *p*_adj_ = 0.004, *η*²_p_ = 0.22, with iRBD patients showing higher surface FA compared to HCs, *t*(62) = 4.2, *p*_adj_ < 0.001, and ePDs, *t*(43) = 4.6, *p*_adj_ < 0.001.

#### PPMI dataset

The main effect of Group in the measure of surface MD in the CM SNc was significant, *F*(2,198) = 8.78, *MSe* = 4.4E-9, *p*_adj_ = 0.04, *η*²_p_ = 0.08, with HCs having lower mean surface MD compared to iRBD patients, *t*(87) = 3.5, *p*_adj_ = 0.002, and ePD patients, *t*(154) = 4.0, *p*_adj_ < 0.001. Again, there was no difference between iRBD patients and ePD patients (Fig. 1b).

### Striatum: mean Bundle MD and FA

#### Local Western dataset

There were no significant effects.

#### PPMI dataset

There were no significant effects.

### Striatum: mean surface MD and FA

#### Local Western dataset

There was a significant main effect of Group in the unparcellated striatum, *H*(2) = 13.2, *p*_adj_ = 0.03, with iRBD patients having higher mean surface MD compared to HCs, *z* = 3.5, *p*_adj_ = 0.03. A significant main effect of Group was observed in the executive, *H*(2) = 14.1, *p*_adj_ < 0.001, rostral motor, *F*(2,85) = 10.6, *MSe* = 1.6E-8, *p*_adj_ = 0.01, *η*²_p_ = 0.20, parietal, *F*(2,85) = 12.0, *MSe* = 9E-9, *p*_adj_ < 0.001, *η*²_p_ = 0.22, and occipital subregions, *H*(2) = 18.0, *p*_adj_ < 0.001. iRBD patients had higher mean surface MD compared to HCs in the executive, *z* = 3.8, *p*_adj_ < 0.001, rostral motor, *t*(62) = 3.1, *p*_adj_ = 0.01, parietal, *t*(62) = 4.5, *p*_adj_ < 0.001, and occipital subregions, *z* = 4.2, *p*_adj_ < 0.001. iRBD patients also had higher mean surface MD compared to ePD patients in the rostral motor, *t*(43) = 4.6, *p*_adj_ < 0.001, and parietal subregions, *t*(43) = 4.3, *p*_adj_ < 0.001.

#### PPMI dataset

There were no significant effects.

### CM SNc and total SNc mean surface MD RF classifier models

**Fig. 2:**
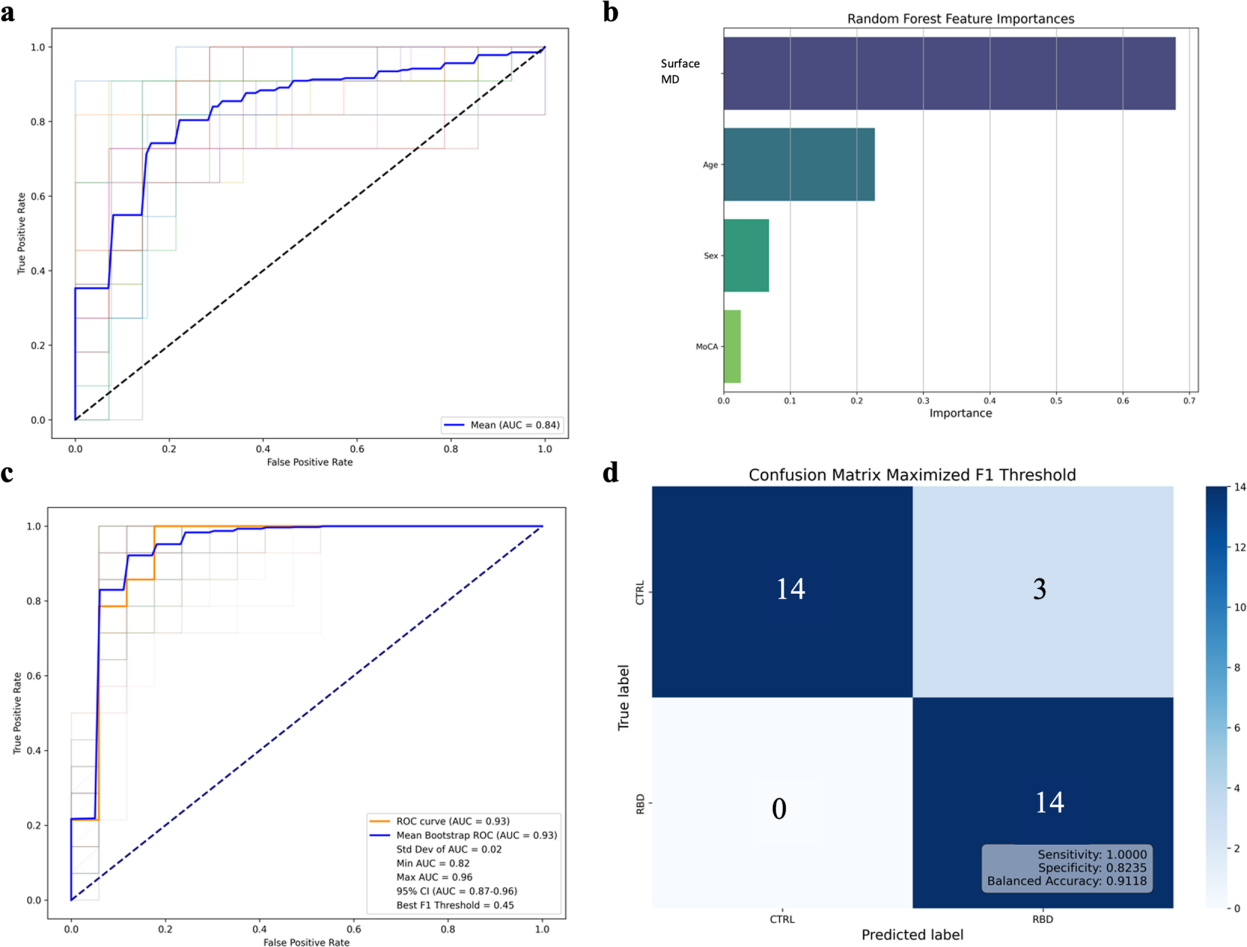
ROC-AUC analysis and confusion matrix for classification of iRBD versus HC using the CM SNc mean surface MD. **a** ROC curves for fivefold cross-validation and the mean AUC score, illustrating performance across different training subsets. **b** Bar chart displaying the relative importance of features as determined by a Random Forest classifier. The Mean surface MD in the CM SNc has the highest importance, followed by Age, MoCA total score, and Sex. **c** ROC curve depicting the fitted model: orange represents the hold-out test set (AUC = 0.93), and blue represents the mean ROC curve from 200 bootstrap resamples of the training dataset (mean AUC = 0.93). The faded lines around the mean bootstrap ROC curve represent the 200 resamples with a calculated 95% confidence interval (CI) for the AUC, ranging from 0.82 to 0.96. Overall, the variation in the bootstrap resample ROC curves (faded lines) indicates the stability of the model’s performance across different subsamples, with a minimum AUC of 0.82 and a maximum AUC of 0.96. The best F1 threshold was 0.45. **d** Confusion matrix that maximises the F1 score on a hold-out test set of 31 cases, with a sensitivity of 1.00, specificity of 0.82, and balanced accuracy of 0.91. Local Western: n_RBD_ = 19, n_HC_ = 45, PPMI: n_RBD_ = 48, n_HC_ = 41.

ROC curve analyses of the CM SNc mean surface MD as an RF classifier, in a model with age, sex and MoCA total scores, was performed on training data for iRBD (Western and PPMI combined) versus HCs. This model revealed good diagnostic accuracy with a mean AUC of 0.84 across fivefolds for validation and hyperparameter optimisation (Fig. 2a). RF feature importance in our model revealed the greatest contribution from CM SNc surface MD in the classification of groups, followed by Age, Sex, and MoCA total score (Fig. 2b). Evaluating generalisability of the model, ROC curve analysis in the 20% randomly-selected, group-stratified, hold-out test data showed excellent diagnostic accuracy, AUC of 0.93 (Fig. 2c) and balanced accuracy of 0.91 (i.e., mean of sensitivity which relates to accuracy classifying cases and of specificity which relates to accuracy classifying controls that were 1.00 and 0.82, respectively; Fig. 2d)]. To further ensure that the hyperparameter-optimised model was representative of the dataset, it was tested in 200 bootstrap resamples with replacement of the training set, mean AUC = 0.93.

No significant iRBD-PD differences were replicated across datasets, and hence there were no potential iRBD-to-PD phenoconversion biomarkers. We next, performed post-hoc analyses to evaluate the *equivalence* of the CM SNc mean surface MD values between iRBD and ePD patients. The aim was to determine if our patient groups could be combined to enable model development in larger datasets, but more importantly, allowing testing of our optimised model in a larger 20% independent hold-out set (*n* = 42 rather than *n* = 14 and *n* = 28 separately). In frequentist statistical analysis, failing to reject the null hypothesis cannot be interpreted as equivalence between Groups or Conditions, because the probabilities of Type II errors (i.e. falsely failing to reject the null hypothesis) are related to the power of studies (i.e. power = 1 - β, where β is the Type II error rate), which is not under the full control of the experimenter, varying in unknown magnitude and determinants in each study^43^. In contrast, in Bayesian analysis, the errors associated with the null and alternative hypotheses are treated in a symmetrical manner. The relative fit of the data to the models representing the null and alternative hypotheses can be directly contrasted^43^. As we have done in previous studies^44^, toward determining the statistical equivalence of the CM SNc mean surface MD values in the iRBD and ePD patients, we performed Bayesian ANCOVAs, with Sex and Age as covariates. In Bayesian analysis, the Bayes Factor in support of the alternative hypothesis (BF10) varies between 0 and ∞. BF10 = 1 signifies the equivalence of null and alternative. BF10 < 1 supports the null hypothesis. BF10 > 1 supports the alternative hypothesis. Our Bayesian ANCOVAs revealed BF10 = 0.507 in the Western data and BF10 = 0.196 in the PPMI data, supporting the null hypothesis in both cases, indicating that iRBD and ePD were statistically equivalent in CM SNc mean surface MD values.

**Fig. 3:**
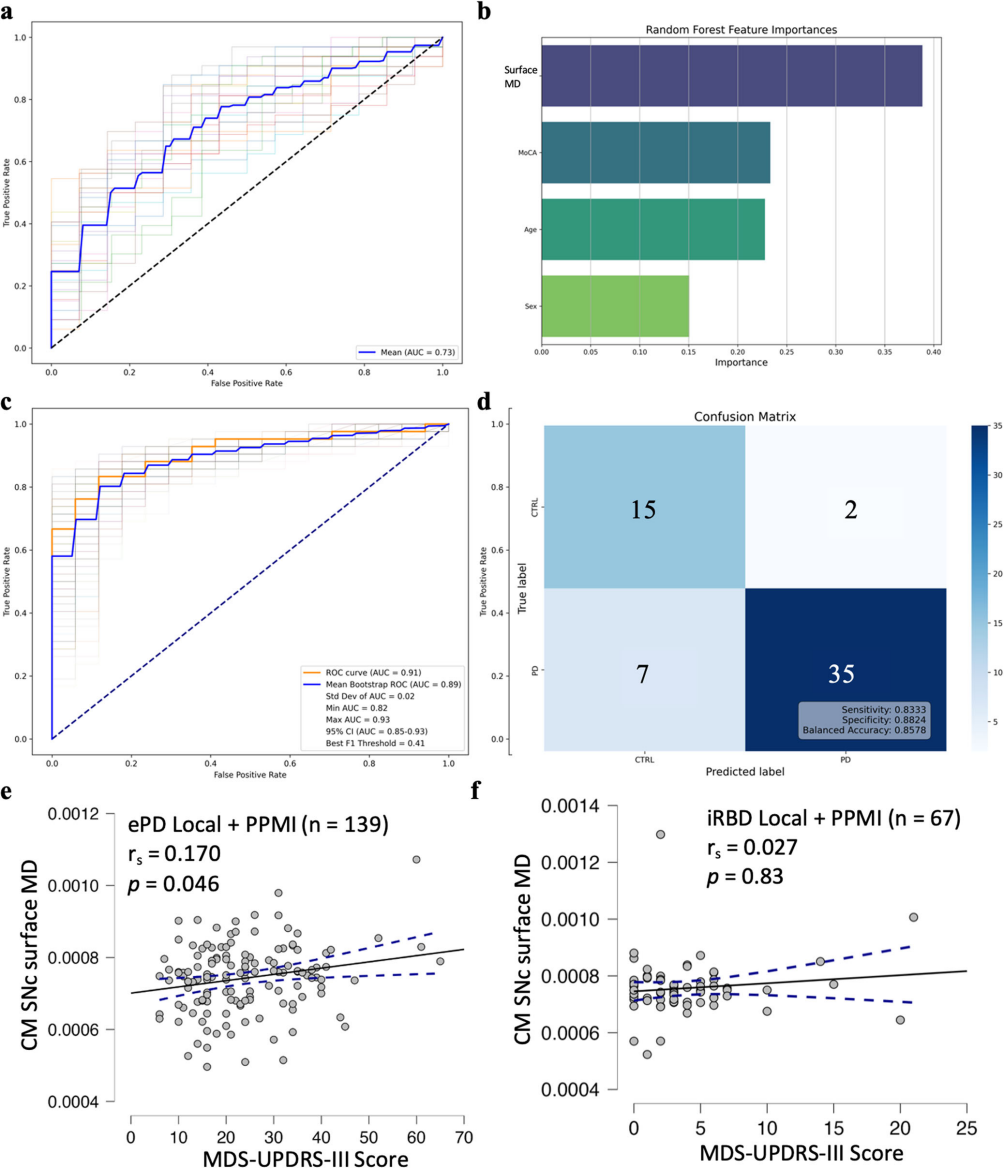
ROC-AUC analyses classifying iRBD and ePD versus HC using CM SNc mean surface MD, and CM SNc mean surface MD-MDS-UPDRS III correlations. **a** ROC curves for fivefold cross-validation and the mean AUC score, illustrating performance across different training subsets. **b** Bar chart displaying the relative importance of features as determined by a Random Forest classifier. The mean surface MD in the CM SNc has the highest importance, followed by Age, MoCA total score, and Sex. **c** ROC curve depicting the fitted model: orange represents the hold-out test set (AUC = 0.91), and blue represents the mean ROC curve from 200 bootstrap resamples of the training dataset (mean AUC = 0.89). The faded lines around the mean bootstrap ROC curve represents the 200 resamples with a calculated 95% confidence interval (CI) for the AUC, ranging from 0.82 to 0.93. Overall, the variation in the bootstrap resample ROC curves (faded lines) indicates the stability of the model’s performance across different subsamples, with a minimum AUC of 0.82 and a maximum AUC of 0.93. The best F1 threshold was 0.41. **d** Confusion matrix that maximises the F1 score on a hold-out test set of fifty-nine cases, with a sensitivity of 0.83, specificity of 0.88 and balanced accuracy of 0.86. **e** Shows the significant positive correlation between CM SNc surface MD and MDS-UPDRS-III motor scoresin ePD patients from Western and PPMI with dashed lines showing the 95% confidence interval. **f** No significant correlation between CM SNc mean surface MD-MDS-UPDRS-III motor scores was observed in iRBD patients from Western and PPMI data combined. Local Western: n_RBD_ = 19, n_ePD_ = 26, n_HC_ = 45, PPMI: n_RBD_ = 48, n_ePD_ = 115, n_HC_ = 41.

We grouped iRBD and ePD, for Western and PPMI combined, for classification assessment against HCs in an RF classifier of CM SNc mean surface MD that included Age, Sex, and MoCA total score as covariates. ROC curve analyses of the training data revealed fair diagnostic accuracy, with mean AUC of 0.73 for model hyperparameter training through fivefold cross-validation (Fig. 3a). RF feature importance again suggested CM SNc mean surface MD contributed most to the classification, followed by Age, MoCA total score, and Sex (Fig. 3b). ROC curve analyses of the RF model in the 20% independent test data revealed excellent diagnostic accuracy, AUC = 0.91 and balanced accuracy = 0.86 (sensitivity = 0.83; specificity = 0.88; Fig. 3d). For the 200 bootstrap resamples of the training data, mean AUC was 0.89 (Fig. 3c).

In contrast, models with total SNc mean surface MD performed more poorly (e.g. balanced accuracy of 0.72; Supplementary Fig. 1).

### Spearman’s correlation analyses

Spearman’s correlation indicated a significant positive relationship, between the MDS-UPDRS-III scores and the CM SNc mean surface MD in the combined Western and PPMI ePD, r_s_ = 0.170, *p* = 0.046 (Fig. 3e), but not for the iRBD, r_s_ = 0.027, *p* = 0.83, patients (Fig. 3f). There were no significant correlations between the CM SNc mean surface MD and (a) the RBDQ-HK scores in local Western iRBD patients, r_s_ = 0.23 p = 0.40, (b) the RBDSQ scores in PPMI iRBD patients, r_s_ = 0.15, *p* = 0.31, or disease duration in local Western iRBD patients, r_s_ = 0.12, *p* = 0.61 (Supplementary Fig. 2). Lastly, Spearman’s correlation analyses revealed a significant positive relationship between the unparcellated SNc mean surface MD and MDS-UPDRS III scores in the combined Western and PPMI ePD patients, r_s_ = 0.19, *p* = 0.03, but this correlation was not significant in iRBD patients, r_s_ = −0.02, *p* = 0.86 (Supplementary Fig. 3).

## Discussion

We investigated whether dopaminergic midbrain nuclei (i.e. the SNc and VTA) and/or the striatum are impaired in iRBD, a prodromal condition of PD, and whether microstructural changes are detected using 3T dMRI in iRBD and ePD. Furthermore, we evaluated whether segmenting midbrain nuclei and striatum, using our automated tractography-based parcellation approach, improved sensitivity to iRBD and/or ePD-related changes using MD and FA measures of white-matter bundles and subcortical grey-matter surfaces. This segmentation enables focussed measurement of SNc and striatal *sub*regions that are affected earliest in PD. Finally, we assessed whether dMRI measures that were significant in group-level analyses across both Western and PPMI datasets, were promising as (a) diagnostic biomarkers of iRBD and/or ePD, (b) phenoconversion biomarkers from iRBD-to-PD, or (c) progression biomarkers of PD.

We tested iRBD (Western *n* = 19; PPMI *n* = 47), ePD (Western *n* = 26, disease duration = 1.7 ± 0.03 years; PPMI *n* = 47 disease duration = 0.9 ± 0.01 years PPMI), and HCs (Western *n* = 46; PPMI *n* = 56) with 3T dMRI. Several group-level analyses were significant in the local Western dataset (Table 3). However, only increases in CM SNc mean surface MD replicated in the multicentred PPMI dataset for iRBD-HCs and ePD-HCs contrasts (Table 3). No unparcellated midbrain or striatal measures were significant in both datasets. Increased CM SNc mean surface MD in iRBD and ePD, reflects abnormal microstructural integrity in the subregion of the SNc that overlaps the nigrosome-1. This is the region predicted to degenerate first in the nigrostriatal system in PD. This measure was sufficiently robust and reliable that it was not obscured by variances related to different scanners and sites in the multicentred PPMI data^40,45^, even in iRBD patients—a prodromal form of PD^45,46^. This measure was not significantly different between iRBD and ePD patients using both frequentist and Bayesian statistical approaches. No other measure was significantly different between iRBD and ePD patients in Western or PPMI datasets, and hence we found no dMRI candidate measures of *phenoconversion* from iRBD-to-PD.

Using CM SNc mean surface MD, with Age, Sex, and MoCA total score as covariates, in the Western and PPMI datasets combined, (a) iRBD patients and (b) iRBD patients and ePD patients combined were distinguished from HCs at the single-subject level with excellent diagnostic accuracies: AUC of 0.93 and 0.91 respectively (Figs. 2c, 3c), and balanced accuracies, which considers both sensitivity and specificity, of 0.91 and 0.86 respectively, in the 20% randomly-selected, group-stratified, independent hold-out sets (Figs. 2d, 3d). To further ensure that the hyperparameter-optimised models were representative, they were tested in 200 bootstrap resamples with replacement of the training data, resulting in mean AUC of 0.93 (iRBD-HCs) and 0.89 (iRBD- and ePD-HCs; Figs. 2c, 3c). In contrast, using the unparcellated SNc mean surface MD in a model including Age, Sex, and MoCA total scores as covariates, classification of iRBD and ePD patients relative to HCs was performed with only fair-to-good diagnostic accuracy/balanced accuracy: AUC = 0.83/0.72 (Supplementary Fig. 1).

Though CM SNc mean surface MD did not distinguish iRBD and ePD patients, the potential of this measure to function as an objective PD progression biomarker was suggested by its correlation with MDS-UPDRS III, the motor subscale, in the ePD group. There was no relation between CM SNc mean surface MD and MDS-UPDRS III in iRBD patients, likely due to a floor effect on MDS-UPDRS III, which is a known weakness of the MDS-UPDRS III, especially in patients who do not have clinically-significant motor symptoms^39^. The lack of iRBD and ePD differences in the CM SNc mean surface MD could owe to the very early stage of our PD groups (disease duration = 1.7 ± 0.3 years, MDS-UPDRS III = 33.2 ± 2.9 Western; disease duration = 0.9 ± 0.01 years, MDS-UPDRS III = 22.8 ± 1.0 PPMI).

Our results in iRBD patients align with a recent study^31^, showing increased MD in iRBD patients, as well as with previous reports and meta-analyses in PD populations^4,21,46^. Our findings and the small extant literature suggest that the SNc—particularly the CM SNc, which overlaps the nigrosome-1, the region in PD that is first degenerated^15,47,48^—is already impacted in iRBD patients^49,50^. This is despite the fact that these patients are not yet exhibiting clinically significant PD motor symptoms on MDS-UPDRS-III. A prior study found decreased FA at the group level in iRBD patients in the total SN, which includes the SNr, as well as the SNc^30^. In our local Western sample, we also noted decreased bundle FA in the total SNc in iRBD patients, though we did not evaluate the SNr. Furthermore, the CM SNc bundle FA was also reduced in iRBD patients, but this was not replicated in the multicentred PPMI dataset.

The failures to replicate dMRI findings in PPMI patients likely owe to the multicentred nature of that dataset, with few participants from each contributing site (Table 4). It is known that quantitative measures of dMRI can vary significantly between scanners/sites^51^^,52^. MD seems systematically more reproducible than FA across the entire brain anatomy in older adults, based on previous work^37^ and might be more robust to scanner effects. In a test-retest multiparametric analysis, Luque Laguna and colleagues noted that MD requires substantially fewer participants than FA to detect the same percentage change in the respective metric longitudinally, except for in the corpus callosum^37^. Here, we found significant group-level differences in seven MD features and only five FA features, which seems to align with this conclusion. All significant differences in iRBD and ePD patients revealed converging trends with higher MD or lower FA for patients compared to HCs, which cohere in suggesting reduced microstructural integrity in iRBD and ePD patients. This is in line with expectations from a systematic review of the PD literature^21^, given that iRBD is a PD-prodromal condition. Though the exact mechanisms remain unknown, increased MD reflects greater overall diffusion, potentially due to cell loss, whereas lower FA reflects decreased tract coherence and increased axonal loss^49^.

**Table 4:** PPMI and Western scanning data stratified across the various sites

Site	RBD	ePD	HC	Total	Manu	Model	Grad Dir	PE Dir
1	0	3	0	3	Siemens	Prisma Fit	30	LR/RL
4	1	1	1	3	Philips	Ingenia	32	LR/RL
7	0	7	3	10	Siemens	TrioTim	64	AP
9	0	1	0	1	Siemens	Prisma	30	RL
12	0	3	0	3	Siemens	TrioTim	64	AP/PA
16	0	1	0	1	Siemens	TrioTim	30	LR/RL
17	4	2	0	6	Siemens	Prisma Fit	30	LR/RL
28	0	2	1	3	Siemens	TrioTim	64	AP/PA
29	0	2	0	2	Siemens	Skyra	30	LR/RL
32	0	3	1	4	Siemens	TrioTim	64	AP/PA
33	0	3	1	4	Siemens	Prisma	30	LR/RL
34	4	3	0	7	Siemens	Prisma Fit, Skyra	30	LR/RL
40	1	1	1	3	Siemens	TrioTim	30	AP
73	2	10	6	18	Siemens	TrioTim, Skyra	64, 30	AP, LR/RL
86	0	1	0	1	Siemens	Biograph	64	AP
88	12	9	2	23	Siemens	TrioTim, Prisma Fit	64, 30	AP, LR/RL
89	0	1	0	1	GE	SIGNA Architect	30	LR/RL
96	0	0	3	3	Siemens	Prisma Fit	30	LR/RL
108	4	2	0	6	Siemens	Skyra	30	LR/RL
120	1	6	2	9	Siemens	TrioTim	64	AP
168	3	2	0	5	Siemens	Prisma Fit	30	LR/RL
196	0	6	0	6	Siemens	Verio	64	AP
289	0	14	2	16	Siemens	TrioTim	64	AP
290	7	11	11	29	Siemens	TrioTim, Prisma Fit	64, 30	AP, LR/RL
291	0	3	3	6	Siemens	Verio, Skyra	64, 30	AP, LR/RL
304	0	1	1	2	Siemens	Verio	64	AP
307	1	3	0	4	Siemens	TrioTim	30	LR/RL
334	0	1	0	1	Siemens	Prisma	64	AP
401	4	10	1	15	Philips	Achieva dStream	32	LR/RL
402	4	0	2	6	Siemens	Prisma	30	LR/RL
403	0	2	0	2	Siemens	Skyra	30	LR/RL
404	0	1	0	1	Siemens	Prisma	30	LR/RL
Western	19	26	45	90	Siemens	Prisma Fit	95	AP/PA
Total	67	139	86	294				

Columns show scanning site, number of scans for RBDs, ePDs, HCs, and total, along with manufacturer, model, gradient directions (Grad Dir), and phase encode direction (PE Dir; LR/RL are left and right, AP/PA are anterior and posterior directions).

We confirmed our second prediction that isolating *subregions* of subcortical structures would improve the sensitivity and accuracy of dMRI measures in iRBD and ePD. Segmenting the SNc increased the sensitivity of dMRI to distinguish patients from HCs (Table 3). No group-level iRBD-HCs or ePD-HCs contrasts were significant across datasets in the unparcellated VTA or SNc. Most studies have failed to reveal abnormalities such as elevated iron or MD, or reduced neuromelanin or FA, in the total VTA or SN/SNc^33,50,53^ in iRBD, with only a minority revealing such abnormalities^30,31,34,35^. This finding seems meaningful given that this corresponds to the region of the nigrosome-1 that is predicted a priori to be most vulnerable to PD pathophysiology, based on information from autopsy^15,54^, DaT imaging^31,55^, and neuromelanin/susceptibility-weighted MRI in PD^56–58^. Abnormalities in the nigrosome-1 in iRBD patients, however, have been detected in less than 50% of patients^53,59^, and have not been significant at the group level in previous studies^53^. Parcellation of midbrain nuclei, however, enabled subtle pathophysiological changes known to occur in ePD to be detected in vivo with dMRI in iRBD patients with a high level of accuracy at the single-subject level, even in an independent sample, in our study.

In PD patients, we have shown previously^9,18^ reduced volume and increased iron, respectively, in the CM striatum. In the current study, no MD or FA dMRI changes were noted for PD patients in the striatum in the Western and PPMI datasets. Potentially accounting for this, PD patients in the current study were at an earlier stage of disease than in our previously published studies (disease duration = 1.7 ± 0.3 and 0.9 ± 0.1 versus 2.4 ± 0.4 and 6.48 ± 0.74). In line with this interpretation, no iron changes in the CM striatum were observed for iRBD patients in our previous study^18^. Alternatively, changes in volume and iron precede changes in dMRI measures. At odds with this, however, in the Western dataset, surface MD was increased in several striatal subregions and even in the total striatum for iRBD patients relative to HCs, though this did not replicate in the PPMI dataset. Further research will be needed to resolve these discrepancies but as others have shown^33,53^ subregional analysis of subcortical regions improves the sensitivity of MRI measures to investigate PD pathophysiological changes and to provide biomarkers.

Toward our final aim, the CM SNc mean surface MD showed promise as a *diagnostic biomarker* of prodromal PD and ePD. Not all iRBD/RBD patients constitute prodromal-PD or patients who will develop another neurodegenerative alpha-synucleinopathy, such as diffuse Lewy body dementia (DLB) or multiple systems atrophy (MSA)^39,40^. Hence, validated diagnostic biomarkers of prodromal PD and/or DLB, or MSA could facilitate recruitment of patients into clinical trials at stages when disease-modifying potential of therapies is anticipated to be maximal due to greater neural substrate to protect^26,39^. Accurate diagnosis of prodromal PD/ePD and recruitment into studies will increase the power of clinical trials to detect true effects. Furthermore, improved diagnostic confidence of PD will lead to earlier referral of these patients for inclusion in interventional studies. Again, it is expected that earlier intervention will increase the efficacy of disease modifiers with more neurons and neural architecture to preserve^39^. Using ROC curve analyses, the RF classifier using the CM SNc mean surface MD with Age, Sex, and MoCA total score as covariates, performed with excellent diagnostic accuracy/balanced accuracy, sensitivity, and specificity in classifying (a) iRBD patients from HCs (AUC = 0.93/0.91; sens = 1.0, spec = 0.83) and (b) iRBD and ePD from HCs (AUC = 0.91/0.86; sens = 0.83, spec = 0.88), at the single-subject level. The utility of dMRI metrics as biomarkers of iRBD and ePD has been questioned given some recent reviews and meta-analyses^60,61^. Our study addresses these uncertainties, and further informs which connectivity features have the greatest potential to act as a biomarker, or to be included in multivariate biomarkers in iRBD and ePD. Furthermore, highlighting how targeting most PD-susceptible subregions improves performance, the unparcellated SNc mean surface MD discriminated iRBD and ePD from HCs with only fair-to-good performance (AUC = 0.82/0.72; sens = 0.90; spec = 0.53), yielding similar results to Gaurav and colleagues (2022) who measured neuromelanin in total SNc to distinguish iRBD and ePD patients from HCs^62^.

Diagnostic biomarkers can improve the clinical management of both conditions as well. Given that iRBD patients with prodromal alpha-synucleinopathy portend serious future health consequences (e.g. severe and faster progressing PD, potential DLB or MSA), accurate diagnosis is important given distress related to anticipation of these future conditions. iRBD diagnosis can be challenging, due to confusion with other parasomnias, and variable rates of access to video polysomnography (vPSG)—the gold-standard diagnostic^63^. Furthermore, vPSG cannot distinguish patients with iRBD who have prodromal PD/neurodegenerative alpha-synucleinopathy from those with symptoms due to other causes related to medications, previous environmental exposures, head trauma, or disorders such as autoimmune conditions^63^. A PD-prodromal diagnostic test is needed, not only to appropriately recruit patients into clinical trials but also to determine those patients for whom disease-modifying treatment will be appropriate once therapies are established. It would be problematic to expose asymptomatic patients to medication for a decade—the average latency for iRBD-to-PD/alpha-synucleinopathy phenoconversion—without any potential for long-term benefit^39^. Diagnostic tests of PD are also needed urgently given the complexity of diagnosis, rising numbers of patients due to demographic shifts, increasing life expectancies, and predicted increasing mismatch between PD prevalence and movement disorder neurologist availability^3^. Diagnostic accuracy varies widely across practitioners depending on their training and expertise. Increasingly the diagnosis of PD will fall to generalists.

The CM SNc mean surface MD did not distinguish iRBD and ePD patients. None of our dMRI measures differentiated these groups, and hence, we did not discover any potential iRBD-to-PD phenoconversion *biomarkers*, which could provide useful objective endpoints in clinical trials of disease modifiers. The lack of iRBD and ePD differences could owe to the very early stage of our PD groups (disease duration = 1.7 ± 0.03 years Western; disease duration = 0.9 ± 0.01 years PPMI). Alternatively, the preponderance of evidence suggests that iRBD and PD are manifestations of the same disease along a spectrum, and hence, clear boundaries might be difficult to determine^39^.

The potential of the CM SNc mean surface MD for providing a *PD progression biomarker* was suggested by its correlation with MDS-UPDRS III in the ePD group. Quantitative progression biomarkers would allow smaller patient groups, with shorter follow-up, given lower variability in objective compared to clinical endpoints of PD^39^. PD is a highly heterogeneous condition. Objective measures could expedite the screening of therapies for their potential to prevent/delay PD advance. Only those treatments screened positively could be investigated in costly trials that include clinical endpoints^40^. There was no relation between CM SNc mean surface MD and MDS-UPDRS III in iRBD patients, likely due to a floor effect with the available clinical measure. An objective biomarker that is distinguished in prodromal PD and ePD, as well as tracks PD disease progression could enable rapid screening of potential disease-modifying compounds, at stages of disease when interventions are expected to be more effective.

The current limitations of this study include significantly male-dominant patient samples, compared to balanced HC groups. We found no iRBD-ePD difference and only a modest correlation for ePD CM SNc surface MD with disease severity. We attribute this to our very ePD patients, with only mild symptoms and possibly the understanding that iRBD patients experience more aggressive PD. Furthermore, iRBD is a prodrome of *alpha-synucleinopathies*. Longitudinal follow-up, ongoing at Western and PPMI, is needed to permit the final interpretation of our findings and evaluation of the prognostic potential of any measures. We employed single-atlas segmentation with the CIT168 subcortical atlas^64^. This atlas was developed in a younger cohort (mean age of 35). However, all participants were age-matched, and we used the same atlas across groups, moderating any disadvantages of the atlas. Although segmentation algorithms are available, they have difficulty delineating the SNc boundary, necessary for our work. Currently, no gold-standard approach to probabilistic tractography has been established. There are some limitations: false positive connections due to noise, crossing fibres and partial volume effects, false negatives in low-fibre density regions, and lack of information on directionality and strength of connections. Our CSD model helps tackle issues associated with crossing fibres better than traditional diffusion tensor imaging, however. We also set minimum limits for fibre connections to prevent subregion assignment with low fibre counts, but this approach neglects voting within these voxels (e.g. caudate voxels close to ventricles). Finally, even with the replication and combining iRBD and ePD groups, our sample sizes remained small, particularly in our test sets, warranting replication in larger, preferably multicentred, prospective studies.

In summary, early changes in the nigrostriatal pathway that typify PD—specifically degeneration of the CM SNc that overlaps the nigrosome-1—were detected in iRBD and ePD relative to HCs using dMRI. Segmenting subcortical nuclei using our automated probabilistic tractography to the cortex, enables targeted measures of subregions of the SNc that are most vulnerable to PD pathophysiology. This improves the sensitivity and accuracy of quantitative dMRI measures to identify iRBD and ePD patients across datasets. CM SNc mean surface MD showed promise as a diagnostic biomarker of iRBD and ePD. Correlation with MDS-UPDRS III in ePD warrants further investigation to evaluate its potential to estimate PD progression across a broader disease range. A widely available biomarker of prodromal PD and ePD could accelerate the search for disease-modifying therapies, where none currently exist, and greatly improve the clinical management of at-risk individuals and ePD patients. Given significant disease heterogeneity, valid and reliable biomarkers of PD will likely be multivariate/multimodal. Further investigations in larger, multicentred samples are warranted to confirm the promise of CM SNc mean surface MD and to uncover other objective measures that will truly translate to clinical research and practice.

## Methods

### Participants

#### Local Western dataset

Nineteen iRBD patients (average disease duration 3.4 years ± SEM 0.7), 26 ePD patients (average disease duration 1.7 years ± SEM 0.3), and 46 age-matched HCs participated at Western University (Table 1). All iRBD patients were diagnosed in the Sleep Disorders Clinic at the London Health Sciences Centre (LHSC) based on video polysomnography and diagnostic criteria^65^. The RBDQ-HK, ranging from 0–100 with a diagnostic threshold of 18–19, measured clinical RBD symptoms and severity^66^. ePD patients were within five years of diagnosis and had their diagnoses confirmed by a licenced movement disorders specialist P.A.M. The MDS-Unified Parkinson Disease Rating Scale Part III (MDS-UPDRS-III) was scored by P.A.M. to confirm the absence of PD in non-PD participants^67^ and to assess motor symptom severity in our ePD group. Age-matched HCs were within five years of their age-matched patients. Participants had no co-existing diagnoses of dementia or other neurological or major psychiatric illness^68^. Participants abusing alcohol, prescription, or illicit drugs, or taking cognitive-enhancing medications were excluded. The MoCA was performed on all participants to rule out cognitive impairment, with a total MoCA score of 23/30 being used as a cut off^69,70^. This MoCA cut-off was intended to exclude iRBD patients inclined to develop Lewy Body Dementia (LBD), who often exhibit cognitive symptoms during the iRBD period^71^. We excluded participants with contraindications to MRI. We report demographic, MoCA, and clinical measures for the Western sample in Table 1. All participants provided informed written consent to the protocol before participating according to the Declaration of Helsinki. This study was approved by the Health Sciences Research Ethics Board at Western University.

#### PPMI dataset

Demographic, cognitive, clinical, and imaging data were obtained through the multicentred PPMI from 47 iRBD patients (average disease duration 2.7 ± 1.3 years; ±SEM), 115 ePD patients (average disease duration 0.9 ± 0.1 years), and 56 HCs who had T1-weighted anatomical and dMRI data acquired at their Baseline visit (Tables 2, 3). The REM Sleep Behaviour Disorder Questionnaire (RBDSQ) was used to screen for RBD symptoms with a score of 5/13 being used as the threshold for iRBD^42^. PD patients were scanned at most within 5 years since diagnosis, paralleling our Western sample. MoCA, UPDRS-III, and Hoehn & Yahr stage appear with demographic data for PPMI participants in Table 2^72,73^. We set a lower limit of 55 years of age.

### Data acquisition

#### Local Western data

Participants were scanned on a 3T Siemens MAGNETOM Prisma Fit whole-body scanner at the Centre for Functional and Metabolic Mapping, Western University, London, Ontario, Canada. The scanner had a 32-receiver channel head coil with head position fixation devices installed and a standard body transmit coil was used. A localiser image established the participant’s position. T1-weighted (T1w) anatomical scans were obtained for structural information, registration of dMRI scans and the segmentation of VTA, SNc, and striatum using the CIT168 probabilistic subcortical atlas^64^. T1w anatomical images were acquired with a magnetisation-prepared rapid gradient echo (MPRAGE) sequence [repetition time (TR) = 2300 ms, echo time (TE) = 2.98 ms, flip angle = 9°, Field of View (FoV) = 256 × 256 mm^2^, 159 slices, voxel size = 1 × 1 × 0.9 mm^3^, receiver bandwidth = 160 Hz/Px, acquisition time = 5:35 min].

We accomplished our subcortical parcellation using dMRI and probabilistic tractography, generating imaging features for group-level comparisons. We acquired dMRI scans using an echo-planar imaging sequence (TR = 3800 ms, TE = 88 ms, flip angle = 90°, gradient directions = 95, b1-value of 1000 s/m^2^, b2-value of 2000 s/mm^2^, FoV = 232 × 232 mm^2^, 72 slices, voxel size = 2 × 2 × 2 mm^3^, receiver bandwidth = 1488 Hz/Px, acquisition time = 7:02 min). We acquired two sequences with reversed phase-encoding direction to correct for susceptibility-induced distortions.

#### PPMI data

T1w anatomicals were acquired with a voxel size of 1 × 1 × 1 mm^3^ using MPRAGE sequences. Acquisition parameters varied slightly across sites, and site-specific details can be found in the PPMI database (Table 4). dMRI scans were obtained using spin echo, echo-planar imaging with TR ~10,000 ms, TE ~80 ms, flip angle = 90°, gradient directions = 30–64, b-value of 1000 s/mm^2^, FoV = 256 × 256 mm^2^, ~80 slices, voxel size = 2 × 2 × 2 mm^3^, acquisition time ~8 min. Two sequences with reversed phase-encoding direction were acquired at most sites to correct for susceptibility-induced distortions (Table 4).

### T1w anatomical preprocessing

T1w anatomicals of all participants were skull-stripped using *SynthStrip*^74^ then bias fields for skull-stripped T1w images were corrected using *N4BiasFieldCorrection* from Advanced Normalisation Tools (ANTs, version 2.2; http://picsl.upenn.edu/software/ants).

*SynthSeg*, a deep learning tool was then used to parcellate the whole cortex^75^. Registration between the MNI152NLin2009cAsym template and subject space was then performed with *greedy*^76^ for T1w and *SynthSeg* outputs (2 channels), using the normalised cross-correlation cost function with default parameters^75^. To generate surfaces for the subcortical structures (VTA, SNc, and striatum), we generated meshes in the template space and propagated them to each subject. The CIT168 probabilistic atlas^64^ segmentation was used to generate a triangulated surface in the template space by creating an isosurface at intensity 0.5, and decimating using 25% (removes 25% of vertices) with PyVista^77^. Because of its small size, we customised the approach for the VTA and SNc, by first resampling the images by 200%, creating the isosurface at 0.3, and not performing any decimation.

### dMRI preprocessing

dMRI data were processed using open-source and containerised applications, *snakedwi* and *diffparc*, which use the Brain Imaging Data Structure (BIDS)^78^ and BIDS apps^79^ standards to perform systematised preprocessing, fitting, image registration, and tractography.

dMRI data were preprocessed with denoising using a local principal component analysis method with (dwidenoise from mrtrix3), and correction of ringing artefacts with the *unring* tool^80,81^. Eddy current distortions were corrected using *eddy* (FSL), with the --repol option enabled for outlier replacement^45,82^. If multiple phase-encoding directions were collected, *top-up* was used to correct for susceptibility distortions, with the resulting parameters were fed into *eddy*^83^. For datasets without multiple phase-encoding directions, we performed a registration-based susceptibility distortion correction with *greedy*^76^ on the average b0 image using the T1w image as a reference. This registration was performed using synthesised T1w-contrast images obtained from the S*ynthSR*^84^ tool, to improve registration between the b0 and T1w. Overlay visualisations depicting the skull-stripping and registration were generated to check for failures in each participant. Registration of the final preprocessed diffusion-weighted image to the T1w space was also performed using *SynthSR* to produce images with matched contrast prior to registration. Preprocessed diffusion-weighted image volumes in the T1w space were then used to fit tensors and estimate diffusion tensor metrics using *mrtrix*^51^. The fibre orientation distribution (FOD) reconstruction using constrained spherical deconvolution (CSD) was performed by using an FA-based response function, with the number of spherical harmonics (l_max) chosen automatically based on the number of gradient directions. Tractography was performed from spheres of radius 0.5 mm centred at each vertex, using the iFOD2 algorithm^51^.

**Fig. 4:**
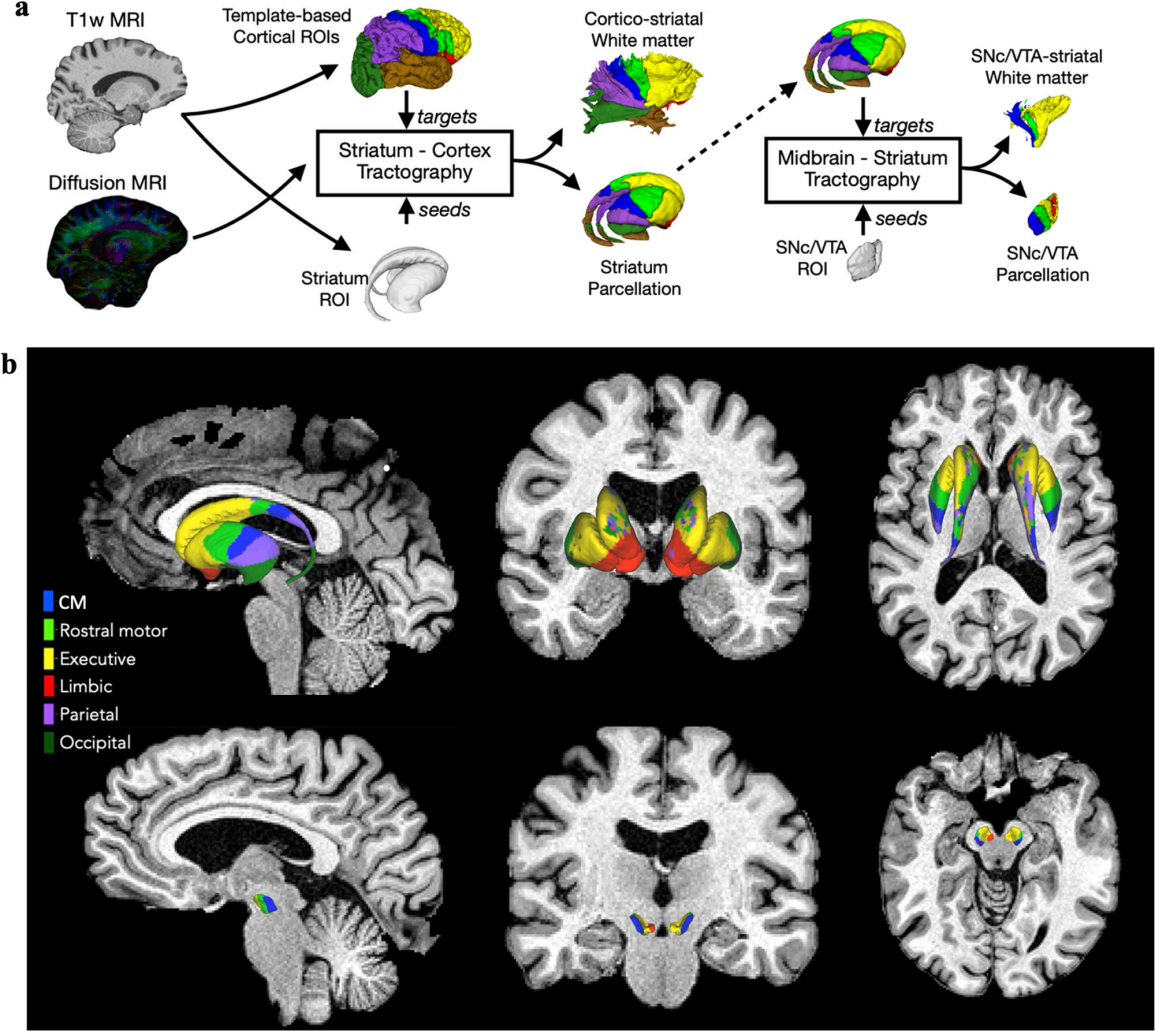
VTA, SNc and striatum parcellation on T1w anatomical of a HC. **a** The CIT168 atlas defines the regions of interest, which are parcellated into subregions according to their tractography-based connection profiles to the cortical subregions. **b** Top row shows the striatum parcellation from probabilistic tractography using the CIT168 atlas in sagittal (left lateral view), coronal (anterior view) and axial (dorsal view) planes with the six subregions in their respective colours. Bottom row shows the VTA and SNc parcellation overlaid on the T1w anatomical in sagittal (left lateral view), coronal (posterior view), and axial (dorsal view) planes.

**Fig. 5:**
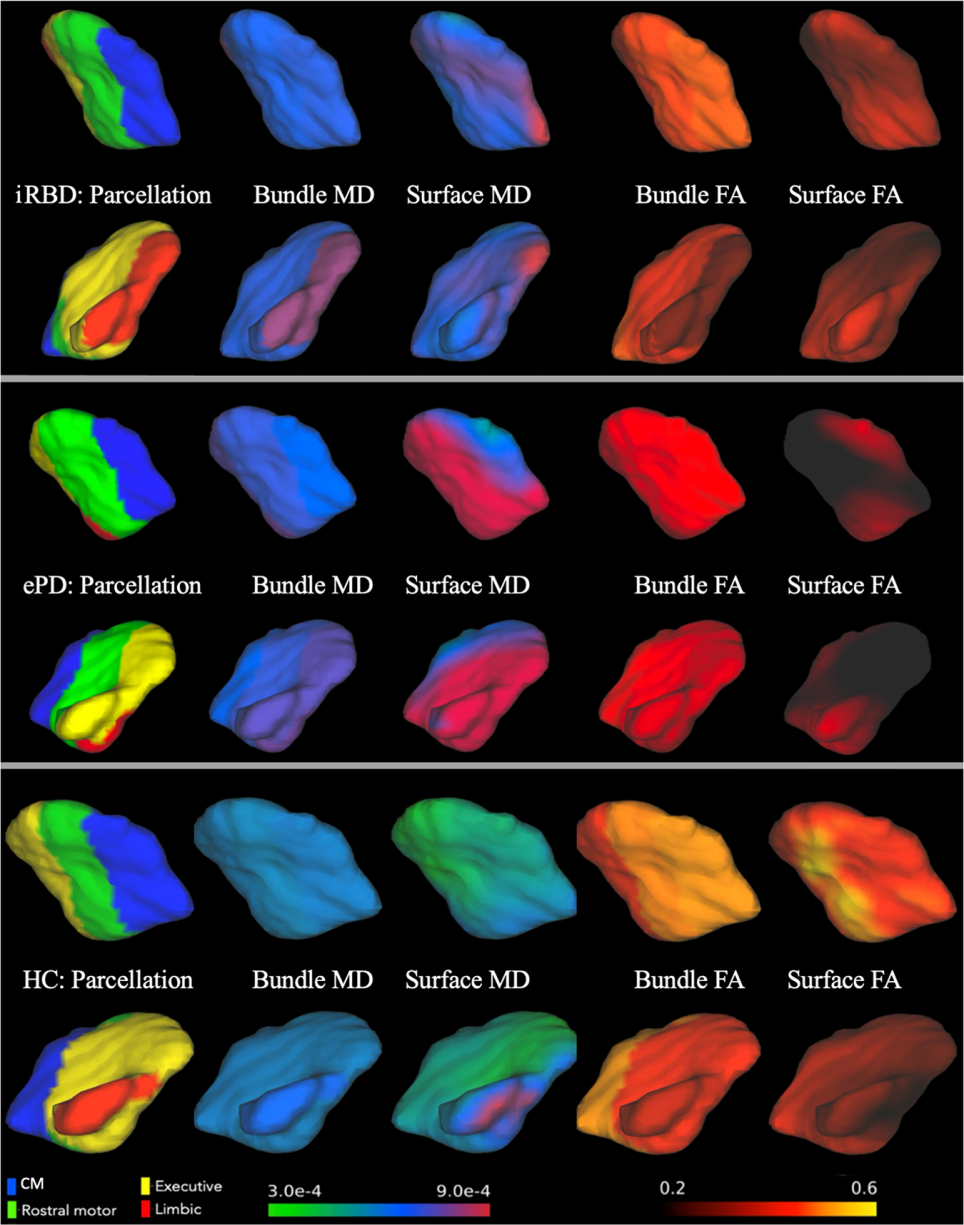
VTA and SNc parcellation of iRBD patient, ePD patient and HC with their dMRI measures. Rows show the left hemisphere VTA and SNc parcellation from lateral and medial views in the sagittal plane with the bundle MD, surface MD, bundle FA and surface FA features of each group of participants.

**Fig. 6:**
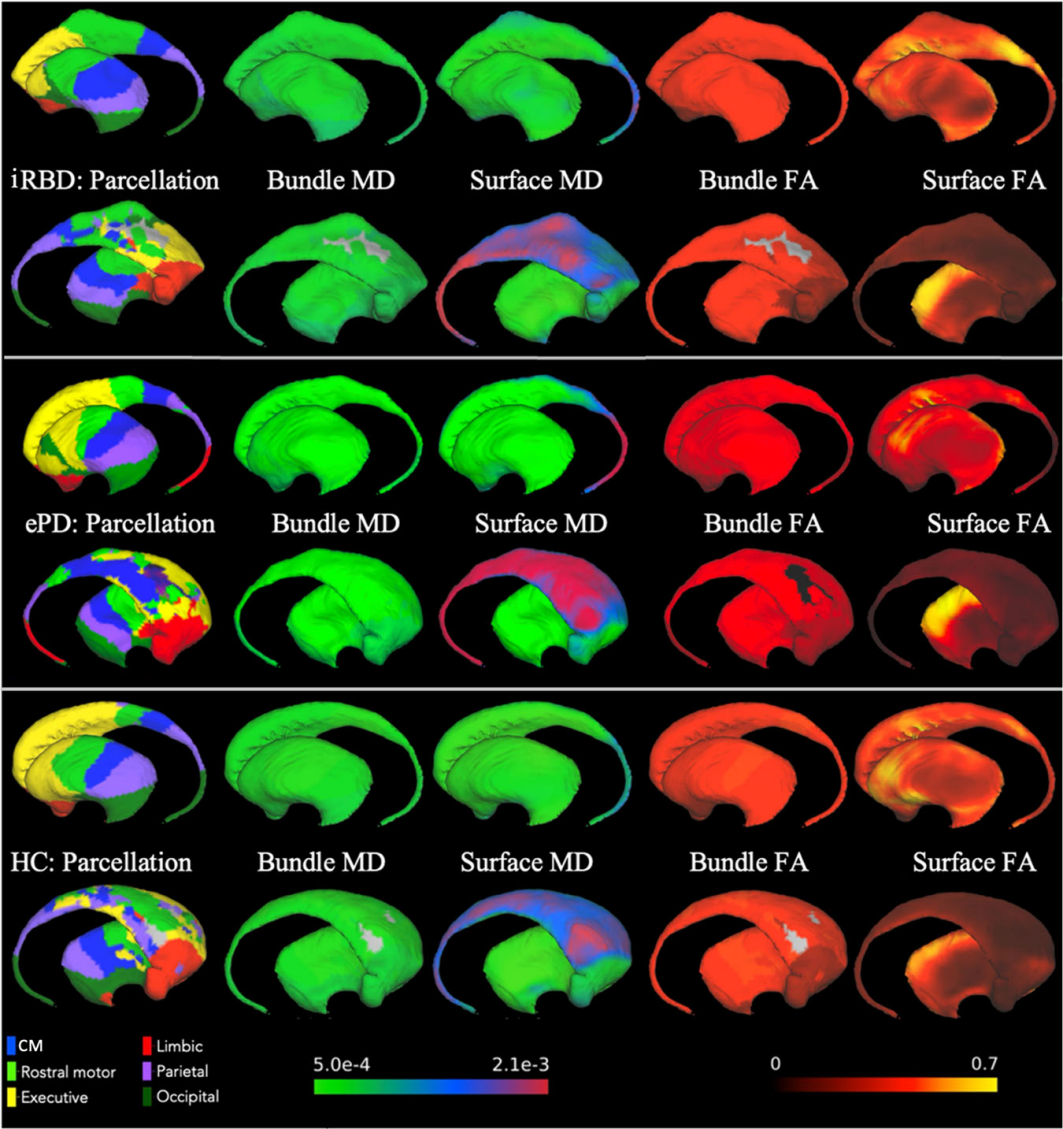
Striatum parcellation of an iRBD patient, ePD patient, and HC with their dMRI measures. Rows show the left hemisphere striatum parcellation from lateral and medial views in the sagittal plane with the bundle MD, surface MD, bundle FA and surface FA features of each group of participants.

Regions of interest in the MNI152NLin2009cAsym template space were chosen to cover the VTA, SNc, and striatum (i.e., caudate nucleus, putamen, and nucleus accumbens) from the recent CIT168 probabilistic subcortical atlas^64^, and cortical labels were built from the Harvard-Oxford atlas, split into six regions in each hemisphere: limbic, CM, rostral motor, executive, parietal and occipital (Fig. 4)^85^. The VTA and SNc were parcellated into four subregions, again based on cortical regions to which they are connected (Fig. 5). As we have done previously, in a subject-specific manner, 250 seeds per vertex on the surface of the striatum were initiated to parcellate the striatum into six subregions, based on the cortical regions to which they were connected (Fig. 6). We also included the entirety of the VTA, SNc, and striatum as a measured of the unparcellated regions to assess the utility of our tractography-based parcellation. All steps in our approach are fully specified or automated, which makes them reproducible across centres and better suited for clinical adoption.

### Bundle MD and bundle FA measures

Bundles are streamlines that originate from the parcels or subregions of the VTA, SNc, or striatum and terminate in the corresponding cortical subregions. These bundles represent the white matter between the vertices of the two subregions (e.g. a bundle exists between the CM striatum and CM cortex). We obtained the bundles using the *tckedit* command in *mrtrix* and then the *tensor2metric* function was used to compute the MD and FA values of the pathways. The bundle volumes masked the MD and FA maps, which permit calculation of the mean MD and FA along the pathways called bundle MD and bundle FA, respectively (Figs. 5, 6).

### Surface MD and surface FA measures

For probing microstructure at the grey/white matter interface, the mean values of the MD and FA maps along the outer surface of the VTA, SNc, and striatum subregions were computed, which corresponded to the surface MD and surface FA features, respectively (Figs. 5, 6).

### Statistical analyses

Demographic, cognitive and clinical data (Age, Education, MoCA total scores, MDS-UPDRS-III, RBD questionnaire scores) were compared across groups using one-way ANOVA. Chi-square was performed for sex. In cases where Levene’s test for equality of variances reached significance, a non-parametric Kruskal–Wallis test (*H*) was used with Dunn’s test (*z*) for post-hoc comparisons. Two iRBD patients, six ePD patients and six HCs from the PPMI were removed from further analyses due to issues with image quality.

Effects of iRBD and ePD on bundle MD and FA and surface MD and FA were assessed in each region of interest in separate analyses of covariance (ANCOVAs) with Group (iRBD, ePD, and HC) as the between-subject factor, controlling for age, sex and site (PPMI data only) as covariates. Kruskal–Wallis test (*H*) with Dunn’s test (*z*) were used for post-hoc comparisons as above. For all statistical analyses, *p* < 0.05 was used as the statistical threshold. Bonferroni correction for multiple comparisons was used to control the family-wise error rate to α = 0.05/96. We also investigated our results with the more liberal Benjamini–Hochberg correction.

Any variables that produced significant group-level differences in both local Western and PPMI data were then further investigated for their potential to classify participants at the single-subject level along with age, sex, and MoCA as covariates in a model developed with the Random Forest (RF) algorithm and evaluated principally using receiver operating characteristic curves (ROC)-area under the curve (AUC) metrics as explained below. Total unparcellated SNc surface MD was also investigated for classification potential with Age, Sex, and MoCA as covariates in an RF model to understand the effect of parcellating the SNc on single-subject classification.

For differences that replicated across samples between iRBD and HCs, or between iRBD and ePD, the RF algorithm, as implemented in the Scikit-Learn Python library was used to construct a classifier for iRBD versus HCs, or for iRBD versus ePD respectively using a) SNc, VTA, or striatal dMRI measures that were significant at the group-level in Western and PPMI samples, and b) total unparcellated SNc mean surface MD, for comparison to parcellated features. Age, sex and MoCA total scores impact brain structures and were used alongside our dMRI features in our model as they had been used in our group-level ANCOVAs. For measures that revealed iRBD versus HCs differences, where iRBD and ePD patients performed equivalently, determined with Bayesian analyses, an RF classifier for RBD and ePD combined versus HCs was developed. During model training on a group-stratified random sample of 80% of the total data, classes were automatically balanced, and a fivefold cross-validation approach was employed, coupled with automated hyperparameter tuning facilitated by the Optuna package [http://optuna.org], which uses Bayesian optimisation, with a probabilistic model called Tree-structured Parzen Estimator (TPE). The TPE model is used to predict the objective value (e.g. AUC accuracy, logloss) of a trial given its hyperparameters, and then Optuna uses this model to suggest new sets of hyperparameters. The range of hyperparameters was initially constrained manually and subsequently optimised within the set boundaries over 100 trials using Optuna. The best hyperparameters were extracted and fit into the final RF model. The key performance metrics of ROC-AUC, balanced accuracy, sensitivity, and specificity were then calculated on the group-stratified, 20% independent hold-out test set. As part of our standard workflow, confusion matrices were generated using the default decision threshold and a threshold that maximises the F1 Score (i.e. a metric that balances sensitivity and precision). In the final step, the training set was resampled, fitted to our optimal model, and assessed against the test set 200 times. All statistical analyses were performed using IBM SPSS Statistics (version 25, IBM Corp., Armonk, NY, USA). Figures were generated using JASP (version 0.17, JASP Team, https://jasp-stats.org/).

Spearman’s correlations were investigated between significant dMRI measures and MDS-UPDRS-III motor scores in (a) iRBD patients from Western and PPMI datasets combined and (b) ePD patients from Western and PPMI datasets combined. We also performed Spearman’s correlations as above, but rather than the MDS-UPDRS III score, we used (a) RBDQ-HK scores in the local Western patients, (b) RBDSQ scores in the PPMI patients, and (c) iRBD disease duration in the local Western patients (Supplementary Fig. 2).

### Supplementary information

Supplementary

## Data Availability

The data that support the findings of this study are available from the corresponding author following a formal data sharing agreement, approval from the requester’s local ethics committee, and submission of a formal project outline.
