# Supplementary material for: Increased mean diffusivity of the caudal motor SNc identifies patients with REM sleep behaviour disorder and Parkinson’s disease
Source: NPJ Parkinsons Dis. 2024 Jun 29;10:128. doi: 10.1038/s41531-024-00731-0 (PMC11217278; doi:10.1038/s41531-024-00731-0)
Supplement: Supplementary file 1 — Supplementary [file 41531_2024_731_MOESM1_ESM.pdf]

Supplementary Material

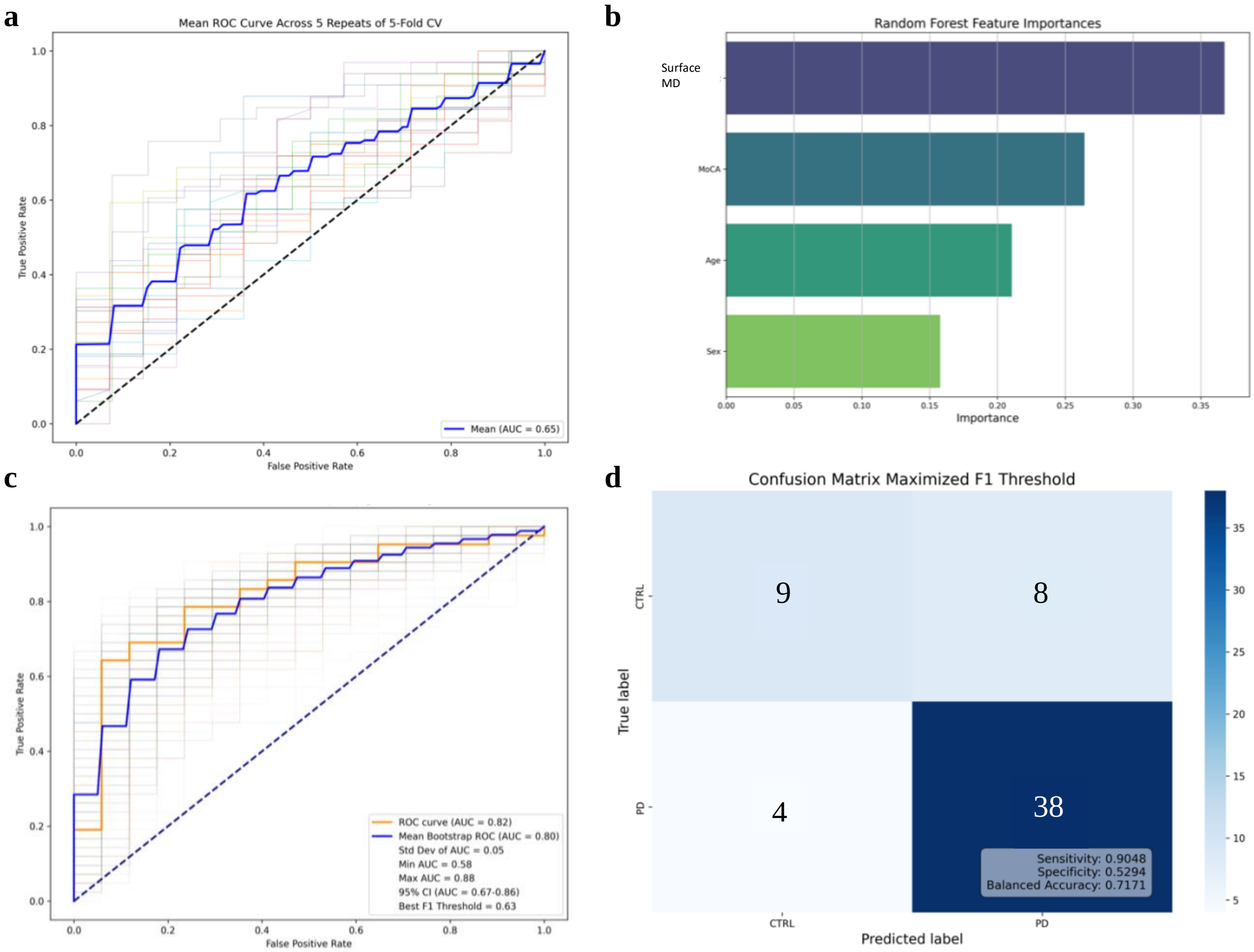

Supplementary Figure 1. ROC Curve analysis and confusion matrix of iRBD and ePD versus HC of the *unparcellated* SNc mean surface MD. a) ROC curves for 5-fold cross-validation and the mean AUC score, illustrating performance across different training subsets. b) Bar chart displaying the relative importance of features as determined by a Random Forest classifier. The Mean surface MD in the unparcellated SNc has the highest importance, followed by Age, MoCA total score, and Sex. c) ROC curve depicting the fitted model: orange represents the hold-out test set (AUC = 0.82), and blue represents the mean ROC curve from 200 bootstrap resamples of the training dataset (mean AUC = 0.80). The faded lines around the mean bootstrap ROC curve represents the 200 resamples with a calculated 95% confidence interval (CI) for the AUC, ranging from 0.67 to 0.86. Overall, the variation in the bootstrap resample ROC curves (faded lines) indicates the stability of the model's performance across different subsamples, with a minimum AUC of 0.58 and a maximum AUC of 0.88. The best F1 threshold was 0.63. d) Confusion matrix that maximizes the F1 score on a hold-out test set of fifty-nine cases, with sensitivity of 0.91, specificity of 0.53, and balanced accuracy of 0.71.
Local Western: $n_{RBD} = 19$, $n_{ePD} = 26$, $n_{HC} = 45$, PPMI: $n_{RBD} = 48$, $n_{ePD} = 115$, $n_{HC} = 41$.

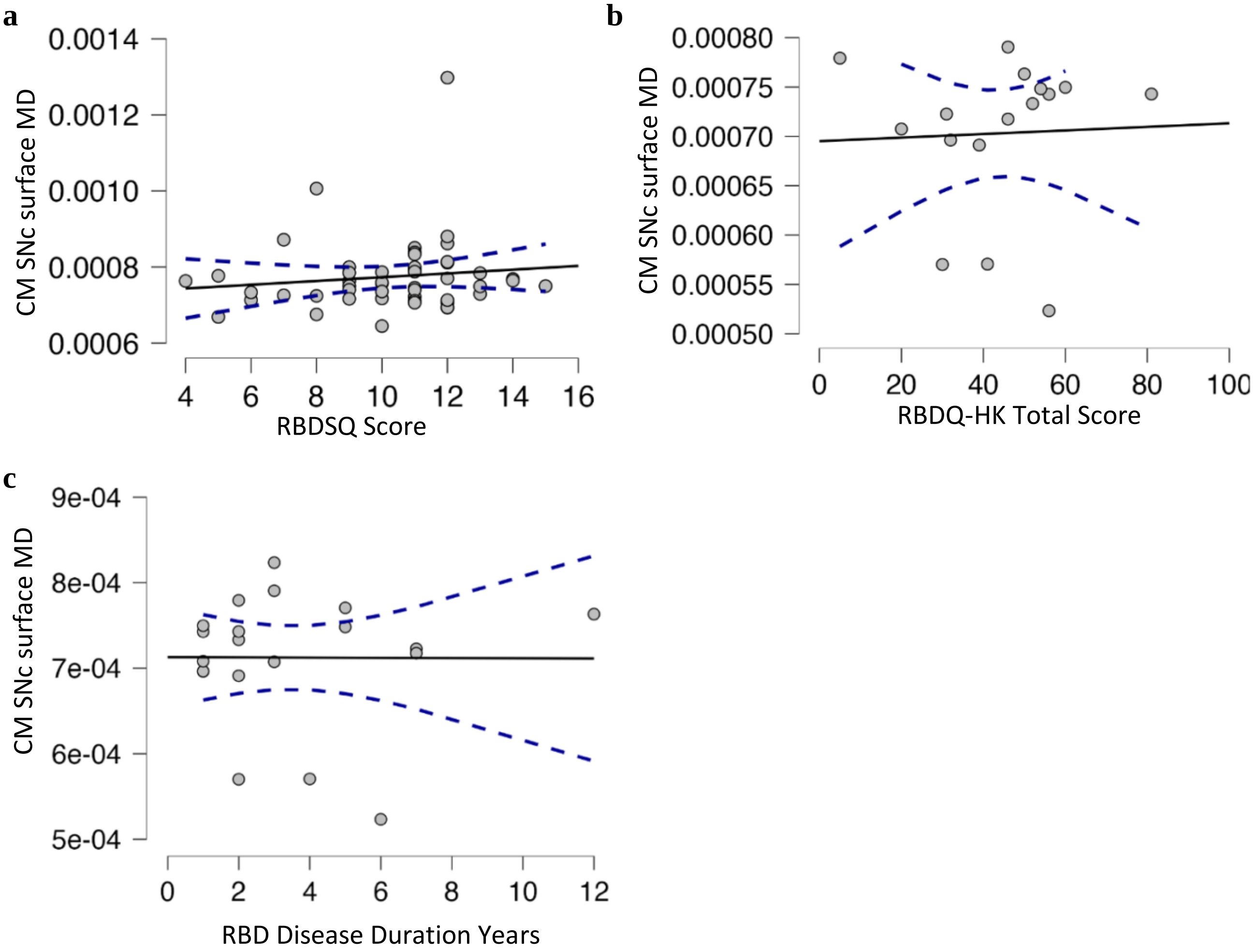

Supplementary Figure 2. Correlations of CM SNc surface MD with a) RBDSQ from PPMI, b) RBDQ-HK from local Western data, and c) RBD disease duration in years from local Western data. a) Spearman's correlation revealed no significant association between CM SNc surface MD and the RBDSQ from the PPMI iRBD patients, $r_s = 0.15$, $p = 0.31$. b) Spearman's correlation revealed no significant association between CM SNc surface MD and the RBDQ-HK from local Western iRBD patients, $r_s = 0.23$, $p = 0.40$. c) Spearman's correlation revealed no significant association between CM SNc surface MD and the RBD disease duration in local Western iRBD patients, $r_s = 0.12$, $p = 0.61$. Local Western: $n_{RBD} = 19$, PPMI: $n_{RBD} = 48$.

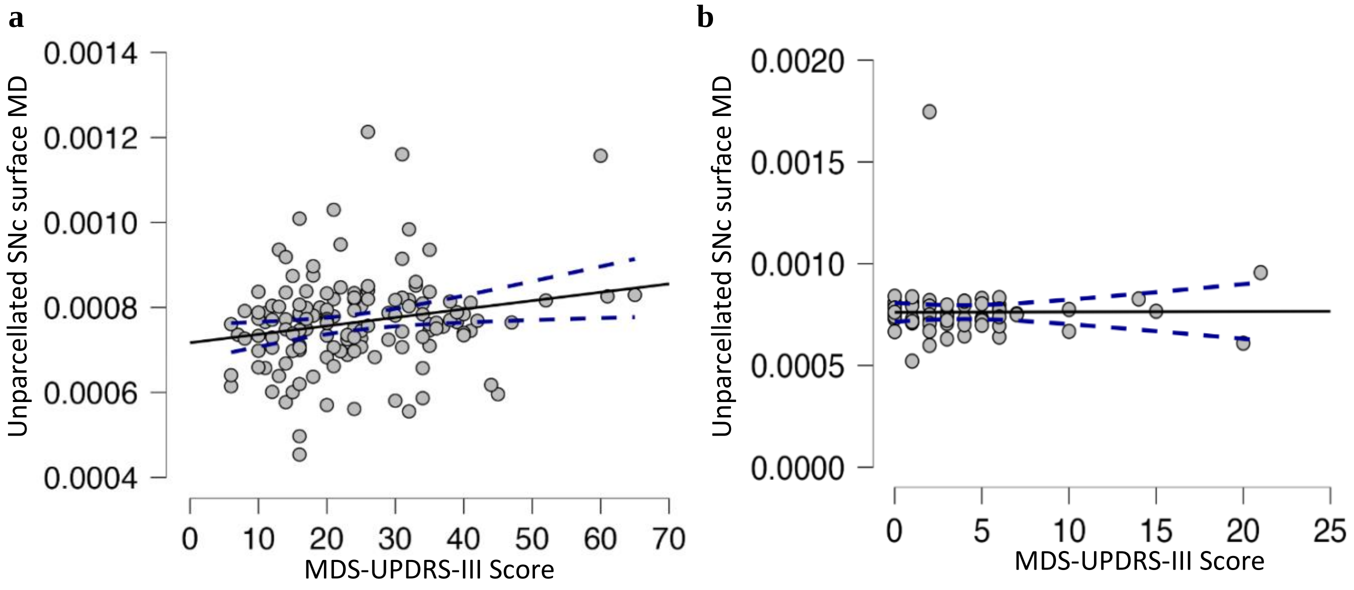

Supplementary Figure 3. Correlation of Unparcellated SNc surface MD and a) ePD patients and b) iRBD patients MDS-UPDRS-III scores in local Western and PPMI datasets. a) Spearman's correlation revealed a significant positive association between Unparcellated SNc surface MD and MDS-UPDRS-III scores in ePD, $r_s = 0.19$, $p = 0.03$. b) Spearman's correlation revealed no significant association between Unparcellated SNc surface MD and MDS-UPDRS-III scores in iRBD, $r_s = -0.02$, $p = 0.86$. $n_{ePD} = 139$, $n_{RBD} = 67$.
